# Application of amide proton transfer imaging for the diagnosis of neonatal hypoxic–ischemic encephalopathy

**DOI:** 10.3389/fped.2022.996949

**Published:** 2022-11-11

**Authors:** Sijin Chen, Xilong Liu, Jie Lin, Yingjie Mei, Kan Deng, Qiao Xue, Xiaoyan Song, Yikai Xu

**Affiliations:** ^1^ Department of Medical Imaging Center, Nanfang Hospital, Southern Medical University, Guangzhou, China; ^2^ Department of Obstetrics & Gynecology, Nanfang Hospital Baiyun Branch, Southern Medical University, Guangzhou, China; ^3^ School of Biomedical Engineering, Southern Medical University, Guangzhou, China; ^4^ C&TS MR Clinical Science, Philips Healthcare, Guangzhou, China; ^5^ Helong Street Community Health Service Center, Guangzhou, China; ^6^ Department of Neonatology, Nanfang Hospital, Southern Medical University, Guangzhou, China

**Keywords:** amide proton transfer, hypoxic–ischemic encephalopathy, neonate, MRI, diagnosis

## Abstract

**Objective:**

This study aimed to evaluate cerebral amide proton transfer signal intensity (SI) among controls, hypoxic–ischemic encephalopathy (HIE) neonates with normal conventional magnetic resonance imaging (HIE/MRI−) findings, and HIE neonates with abnormal conventional MRI (HIE/MRI+) findings.

**Methods:**

Forty neonates diagnosed with neonatal HIE and sixteen normal neonates were evaluated. All neonates underwent conventional MRI scans and APT imaging. Cerebral APT SIs were compared to identify cerebral regions with significant APT changes among sixteen controls, thirteen HIE/MRI− patients, and twenty–seven HIE/MRI+ patients.

**Results:**

Significantly increased APT SIs were observed in the HIE/MRI− group compared with controls, in the left insula, right occipital lobe, left cingulate gyrus (posterior part), and corpus callosum. Significantly increased APT SIs were found in the HIE/MRI+ group compared with controls, in the right anterior temporal lobe (medial part), anterior parts of the right parahippocampal and ambient gyri, left superior temporal gyrus (middle part), left insula, left cingulate gyrus (posterior part), and right lentiform nucleus. No significant APT SI differences were observed in the cerebellum and brainstem among the three groups.

**Conclusion:**

Amide proton transfer imaging plays an important role in detecting hypoxic–ischemic encephalopathy regardless of conventional MRI findings. Changes in APT signal intensity may provide important insights into the characterization of the cerebral internal environment. This study suggests that APT imaging could be used as a complement to conventional MRI in the detection of hypoxic–ischemic encephalopathy in clinical practice.

## Introduction

Neonatal hypoxic–ischemic encephalopathy (HIE) is a major cause of mortality and neurological disability in children. The incidence of HIE is estimated at 3–6 per 1,000 live births (1, 2). It has been reported that therapeutic hypothermia could reduce morbidity and mortality of HIE infants and achieve better neurodevelopmental outcomes (3, 4). However, a high proportion of infants with moderate to severe HIE still show subsequent neurodevelopmental disorders. Therefore, further research on early detection, injury assessment, and neurologic outcome prediction is needed.

Magnetic resonance imaging (MRI) is the most commonly applied imaging method in clinics, evaluating brain injury in neonates with HIE (5). Conventional MRI is less sensitive in detecting likely microstructural injuries than the latest imaging techniques, and done in first days of life may miss significant brain injuries (especially after brain cooling). However, they can help to exclude other causes of encephalopathy (such as congenital malformation, cerebral infarction and hemorrhage). Diffusion–weighted imaging (DWI) and magnetic resonance spectroscopy (MRS) is more sensitive and specific imaging modality to diagnosis acute brain injure. DWI can detect increased signal intensity in acute phase before conventional MRI due to cytotoxic edema (from hypoxia or other causes of cell swelling). The limitation of DWI is that it may give false negative result and underestimate the extent of injury if performed within first 24 h of hypoxic–ischemic injury. And DWI cannot detect the severity of hypoxic–ischemic injury or predict adverse clinical outcome. MRS is very sensitive to the severity of hypoxic–ischemic brain injury and can predict adverse outcome in the first 24 h of life. An elevated ratio of lactate to *N*-acetylaspartate in deep gray matter can predict long–term neurologic impairments [Barta H, Jermendy A, Kolossvary M, et al. Prognostic value of early, conventional proton magnetic resonance spectroscopy in cooled asphyxiated infants. BMC Pediatr. 2018;18(1):302. Published 2018 Sep 15. doi:10.1186/s12887-018-1269-6]. However, these MRI modalities didn't to detect changes in the internal environment in neonatal HIE.

Chemical exchange saturation transfer (CEST) enables the indirect detection of low–concentration endogenous molecules *via* chemical exchange with water protons (6). Amide proton transfer (APT) imaging is the most extensively investigated type of CEST, that is geared towards detecting exchangeable amide protons in the endogenous mobile proteins and peptides in tissue. APT imaging is a promising modality for the relatively non-invasive *in vivo* characterization of the internal environment during neonatal HIE. Although the clinical applications of APT imaging remain in their investigative stages, APT imaging, as a novel pH-weighted imaging technique, has shown excellent performance in the assessment of ischemia, brain tumors, and several other diseases (7).

In a previous study (8), we found neonates with mild HIE have significantly higher APT signal intensities (SIs) compared with healthy neonates in bilateral caudate, bilateral centrum semiovale, bilateral thalamus, and left globus pallidus/putamen. However, not all neonatal HIE patients have abnormal conventional MRI findings. There is a literature gap regarding the evaluation of APT SI changes in HIE neonates with normal conventional MRI findings. In this study, the major aim was to explore cerebral APT SI changes in HIE neonates with normal and abnormal conventional brain MRI findings.

## Materials and methods

### Subjects

This study was approved by the medical ethics review board of the Nanfang Hospital of Southern Medical University, and written informed consent of parents had been obtained before the examinations. (Grant No. NFEC-2021-084).

Fifty–six full–term neonates (gestational age: 36–42 weeks) in the department of pediatrics of Nanfang hospital, Southern Medical University from January 2020 to January 2022 were selected. The inclusion criteria for full–term neonates with HIE were as follows: a history of fetal intrauterine distress or perinatal asphyxia; encephalopathy independently graded by two neonatologists based on modified Sarnat criteria (9). Based on a previously reported scale, HIE was classified as mild, moderate, and severe. Exclusion criteria were: gestational age <36 weeks or >42 weeks; birth weight <2,500 grams or >4,000 grams; convulsions caused by intracranial bleeding; brain injuries caused by genetic metabolic diseases; electrolyte disturbances and birth trauma; intrauterine infection; other congenital diseases. The HIE group was divided into two subgroups: HIE/MRI− and HIE/MRI+. Patients in the HIE/MRI− group had clinical HIE and normal conventional MRI, while those in the HIE/MRI+ group had clinical HIE and abnormal conventional MRI.

Control cases met the following criteria: (1) MRI performed 14 days after birth, without any identified anatomical abnormalities (10); no neurologic conditions, (3) gestational age between 36 and 42 weeks; (4) birth weight was in the range of 2,500–4,000 grams; (5) MRI and APT data available without artifacts.

In all neonates, brain electrical activities were monitored by using an amplitude–integrated electroencephalography (aEEG) machine within 6 h after birth. Sustained normal voltage indicated normal brain activity, while flat waves, burst suppression, discontinuous normal voltage, and continuous low voltage were considered to be abnormal (11). As soon as HIE is clinically diagnosed, prescribed supportive treatment was directly initiated. Moderate or severe HIE infants were treated with hypothermia.

### The Bayley-III scores acquisition

Neurodevelopmental assessments were performed according to the Bayley Scales of Infant and Toddler Development, 3rd Edition (Bayley-III) (12), which provides cognitive, language, and motor composite scores at approximately 6 months, directed by a trained pediatric developmental expert with six years of experience, in collaboration with a neurodevelopmental pediatrician with over 10 years of experience.

### MRI acquisition

All infants were sedated by oral chloral hydrate (10 mg/kg) 20 min before scanning. Scans were performed on a 3 T MRI scanner (Achieva; Philips Healthcare, Best, the Netherlands), using an eight–channel sensitivity–encoding head coil for the reception and a dual–channel body coil for transmission.

All infants underwent T1 weighted imaging (T1WI), T2-weighted imaging (T2WI), diffusion–weighted imaging (DWI), and APT of the brain. For simplicity, T1WI (repetition time: 600 ms; echo time: 10 ms; slice thickness: 4 mm; no gap between slices), T2WI (repetition time: 3,000 ms; echo time: 100 ms; slice thickness: 4 mm; no gap between slices), and DWI (repetition time: 3,463 ms; echo time: 114.3 ms; b factors: 0 and 800 s/mm^2^; slice thickness: 4 mm; no gap between slices) sequences would be referred to as conventional MRI.

All infants were assessed for the severity of HIE-related brain injury based on conventional MRI as previously described. According to the modified Barkovich scale (13), infants were categorized as follows: 0, normal; 1, mild injury (i.e., injury of the periventricular white matter); 2, moderate injury (i.e., injury of the basal ganglia/thalamus or cortex); or 3, severe injury (i.e., injury of the basal ganglia/thalami and cortex). Image analysis was performed by pediatric neuroradiologists with over 10 years of experience.

APT imaging was performed with a fat–suppression, three–dimensional TSE-Dixon pulse sequence. The saturation pulse with a duration of 2 s (40 × 50 ms, sinc–Gauss–shaped elements) and a power level of 2 µT was obtained with two alternated radiofrequency (RF) transmission channels, which enabled long RF irradiation. The saturation frequency offsets (±3.5, ± 3.42, ± 3.58, and −1,540 ppm) were acquired by TSE-Dixon scanning with intrinsic B0 mapping and online reconstruction of B0-corrected APT images. The B0 map was derived from three acquisitions at +3.5 ppm with different echo shifts. The remaining parameters were as follows: repetition time/echo time (TR/TE): 6,835/6.8 ms; field-of-view (FOV): 140 × 119 × 68 mm^3^; voxel size: 1.2 × 1.2 × 4 mm^3^; matrix: 116 × 99; SENSE factor: 2.25; scan duration: 320 s.

### Processing of imaging data

**Figure 1:**
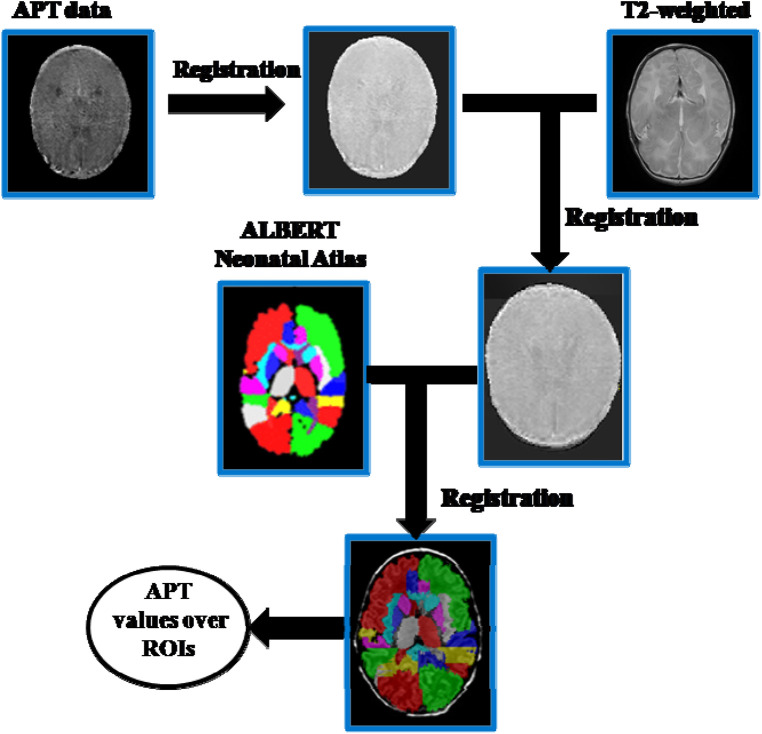
Flowchart of the APT data processing method.

Statistical Parametric Mapping 8 (SPM8) and MATLAB (Mathworks, Inc., Natick, MA) were used for image processing and analysis. APT data preprocessing of neonate images included motion correction, co-registration with the T2-anatomic sequence, smoothing, and brain masking for excluding out-of-brain voxels. The extracted T2 structural data were segmented using the SPM8 toolbox. Then the APTw image was warped into the MNI space using the T2 segmentation–derived transformation matrix. For brain tissue classification, we chose the “ALBERTs atlas” available at https://brain-development.org/brain-atlases/neonatal-brain-atlases/neonatal-brain-atlas-gousias/ rather than using the default SPM8 template (MNI ICMB152) tissue probability maps as an *a priori*. The former automatically segments neonatal brain MR images into 50 ROIs (14). The segmentations provided with this atlas were used to segment the registered APT maps for group analysis (Figure 1).

Magnetization transfer ratio asymmetry (MTR_asym)_ analysis was employed for the calculation of APT SIs. APT weighted values were calculated according to a previously described formula:

$$\mathrm{MT}R_{\mathrm{asym}}\left(\%\right)=\left(S-\Delta \omega -S\Delta \omega \right)/S0,$$

where S-Δ*ω* is B0 corrected signal at −3.5 ppm, SΔ*ω* is B0 corrected signal at +3.5 ppm, and S0 is the normalization factor acquired at −1,540 ppm (15).

### Statistical analysis

SPSS version 22.0 for Windows (SPSS, Inc., Chicago, IL, United States) was used to perform statistical analysis, and measurement data were expressed as mean ± standard deviation (*x* ± *s*).

Between–group comparisons (HIE vs. full–term controls) of birth weight, age in days, gestational age at birth, Apgar score, umbilical artery blood gas parameters, and neurodevelopmental assessment were conducted by independent–samples *t*-test. The *χ*^2^ test was conducted to assess gender, delivery, aEEG, and MRI data between the groups. For multiple–group comparisons, the results of neurodevelopmental assessment scores were compared with the one–way analysis of variance (one–way ANOVA), followed by the Least Significant Difference (LSD) *post hoc* test. The ALBERTs infant brain atlas was adopted to identify brain regions by statistical comparison among the control, HIE/MRI− and HIE/MRI+ groups. The difference with *p* < 0.05 was considered statistically significant.

## Results

### Clinical characteristics

The HIE group included 40 full–term neonates, including 28 males and 12 females. Their gestational ages at birth were in the range of 37–41 weeks, with an average of 39.47 ± 1.19 weeks. Birth weights were 3.25 ± 0.41 kg. Among them, 27 neonates with clinical HIE and abnormal conventional MRI were assigned to the HIE/MRI+ subgroup, and 13 neonates with clinical HIE and normal conventional MRI were assigned to the HIE/MRI− subgroup. Besides, the HIE/MRI+ group consisted of 18 mild cases, 7 moderate cases, and 2 severe cases, while the HIE/MRI− group comprised 13 mild cases. The control group included 16 full–term neonates (9 males and 7 females, gestational ages at birth 39.60 ± 1.07 weeks; birth weights were 3.10 ± 0.38 kg).

The study population comprised 40 full–term neonates with HIE as cases and 16 full–term neonates as controls. No statistically significant differences were found between the two groups in age, gender, birth weight, gestation age at birth, and delivery (*p* > 0.05). The pH of cord blood and Apgar score at 1 min were significantly reduced, while lactate was significantly increased in the HIE group compared with controls. Table 1 summarizes the clinical characteristics of the study participants.

**Table 1:** Demographic and clinical characteristics of neonates with HIE and controls.

	Control group (*n* = 16)	HIE group (*n* = 40)	*p*-values
Age (days)	4.62 ± 2.55	5.10 ± 2.81	0.560
Gender			0.326
Male	9	28	
Female	7	12	
Gestational ages at birth (weeks)	39.60 ± 1.07	39.47 ± 1.19	0.718
Age after birth at scan (days)	3.3 ± 0.86	4.2 ± 1.31	0.957
Birth weight (kg)	3.10 ± 0.38	3.25 ± 0.41	0.205
Delivery			0.158
Transvaginal delivery	8	28	
Cesarean section	8	12	
Apgar at 1 min	8.75 ± 0.58	7.89 ± 1.57	0.005*
Apgar at 5 min	9.87 ± 0.34	9.84 ± 0.49	0.81
Apgar at 10 min	9.94 ± 0.25	9.97 ± 0.16	0.529
Blood glucose	4.57 ± 1.06	5.09 ± 1.61	0.263
Cord blood pH	7.29 ± 0.10	7.20 ± 0.13	0.013*
Lactate	4.27 ± 2.48	6.11 ± 2.88	0.029
aEEG			
Normal	16	29	
Mild abnormal	0	9	
Moderate abnormal	0	2	
Severe abnormal	0	0	
MRI^a^			
0	16	13	
1	0	20	
2	0	7	
3	0	0	

Values are mean ± standard deviation $(\bar{x}+s)$

*n*, number of subjects; HIE, hypoxic–ischemic encephalopathy.

^a^ Grade 0-1-2-3 = stage of brain injuries (normal, mild, moderate, severe, respectively) according to modified Barkovich scale (13).

* *p* < 0.05.

### Cerebral APT SI changes in the HIE and control groups

Compared with controls, APT SIs were significantly increased in the HIE group. Increased APT SIs were observed primarily in the right hippocampus, anterior parts of the right parahippocampal and ambient gyri, left superior temporal gyrus (middle part), left insula, right occipital lobe, left cingulate gyrus (posterior part), right sub-thalamic nucleus, right lentiform nucleus, and corpus callosum (Table 2).

**Table 2:** Brain regions with significant APT signal intensity differences between the control and HIE groups.

Brain regions	Control (*n* = 16)	HIE (*n* = 40)	*p*-value (*p *< 0.05)
right hippocampus	2.95 ± 0.73	3.40 ± 0.77	0.047
right parahippocampal and ambient gyri anterior part	2.85 ± 0.63	3.39 ± 0.82	0.023
left superior temporal gyrus, middle part	2.63 ± 0.66	3.00 ± 0.57	0.038
left insula	2.59 ± 0.76	3.05 ± 0.48	0.008
right occipital lobe	2.99 ± 0.86	3.44 ± 0.59	0.027
left cingulate gyrus, posterior part	2.64 ± 0.77	3.12 ± 0.61	0.016
right sub-thalamic nucleus	3.07 ± 0.71	3.44 ± 0.53	0.038
right lentiform nucleus	2.67 ± 0.68	3.16 ± 0.73	0.027
corpus callosum	2.61 ± 0.82	3.04 ± 0.59	0.034

Values are mean ± standard deviation $(\bar{x}+s)$.

*n*, number of subjects; HIE, hypoxic–ischemic encephalopathy.

Further comparisons between the control and HIE/MRI− groups and between the control and HIE/MRI+ groups were performed. In the HIE/MRI− group, significantly increased APT SIs were detected in the left insula, right occipital lobe, left cingulate gyrus (posterior part), and corpus callosum (Table 3). In the HIE/MRI+ group, significantly increased APT SIs were observed in the right anterior temporal lobe (medial part), anterior parts of the right parahippocampal and ambient gyri, left superior temporal gyrus (middle part), left insula, left cingulate gyrus (posterior part), and right lentiform nucleus (Table 4).

**Table 3:** Brain regions with significant APT signal intensity differences between the control and HIE/MRI− groups.

Brain regions	Control (*n* = 16)	HIE/MRI− (*n* = 13)	*p*-value (*p *< 0.05)
Left insula	2.59 ± 0.76	3.03 ± 0.29	0.043
Right occipital lobe	2.99 ± 0.86	3.60 ± 0.57	0.018
Left cingulate gyrus, posterior part	2.64 ± 0.77	3.23 ± 0.46	0.019
Corpus callosum	2.61 ± 0.82	3.14 ± 0.53	0.037

Values are mean ± standard deviation $(\bar{x}+s)$.

*n*, number of subjects; HIE, hypoxic–ischemic encephalopathy.

**Table 4:** Brain regions with significant APT signal intensity differences between the control and HIE/MRI+ groups.

Brain regions	Control (*n* = 16)	HIE/MRI+ (*n* = 27)	*p*-value (*p *< 0.05)
Right anterior temporal lobe, medial part	1.75 ± 0.89	2.37 ± 0.86	0.025
Right parahippocampal and ambient gyri anterior part	2.85 ± 0.63	3.45 ± 0.88	0.017
Left superior temporal gyrus, middle part	2.63 ± 0.66	3.03 ± 0.65	0.038
Left insula	2.59 ± 0.76	3.06 ± 0.56	0.012
Left cingulate gyrus, posterior part	2.64 ± 0.77	3.06 ± 0.66	0.045
Right lentiform nucleus	2.67 ± 0.69	3.17 ± 0.83	0.032

Values are mean ± standard deviation $(\bar{x}+s)$.

*n*, number of subjects; HIE, hypoxic–ischemic encephalopathy.

Comparing the HIE/MRI− and HIE/MRI+ groups, significantly decreased APT SIs were identified in bilateral anterior temporal lobes (medial part) in the HIE/MRI− group (Table 5). There was no statistically significant difference in the APT measurements between the left and right hemispheres in control.

**Table 5:** Brain regions with significant APT signal intensity differences between the HIE/MRI− and HIE/MRI+ groups.

Brain regions	HIE/MRI+ (*n* = 27)	HIE/MRI− (*n* = 13)	*p*-value (*p *< 0.05)
Right anterior temporal lobe, medial part	2.37 ± 0.86	1.71 ± 0.79	0.027
Left anterior temporal lobe, medial part	2.57 ± 0.87	1.81 ± 0.79	0.017

Values are mean ± standard deviation $(\bar{x}+s)$.

*n*, number of subjects; HIE, hypoxic–ischemic encephalopathy.

### Neurodevelopmental outcomes

**Figure 2:**
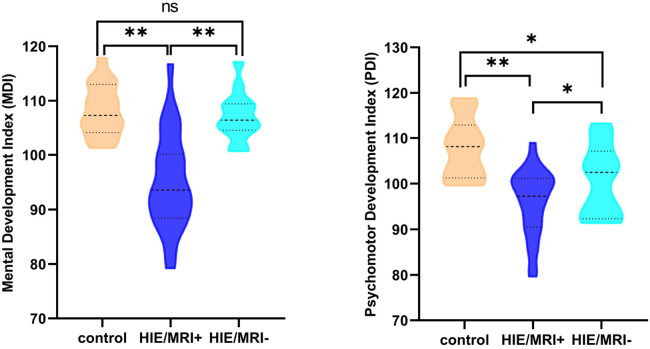
Comparison of MDI and PDI scores in the three groups. A single asterisk indicates *p* < 0.05; double asterisks, *p* < 0.001; ns, no significant.

**Figure 3:**
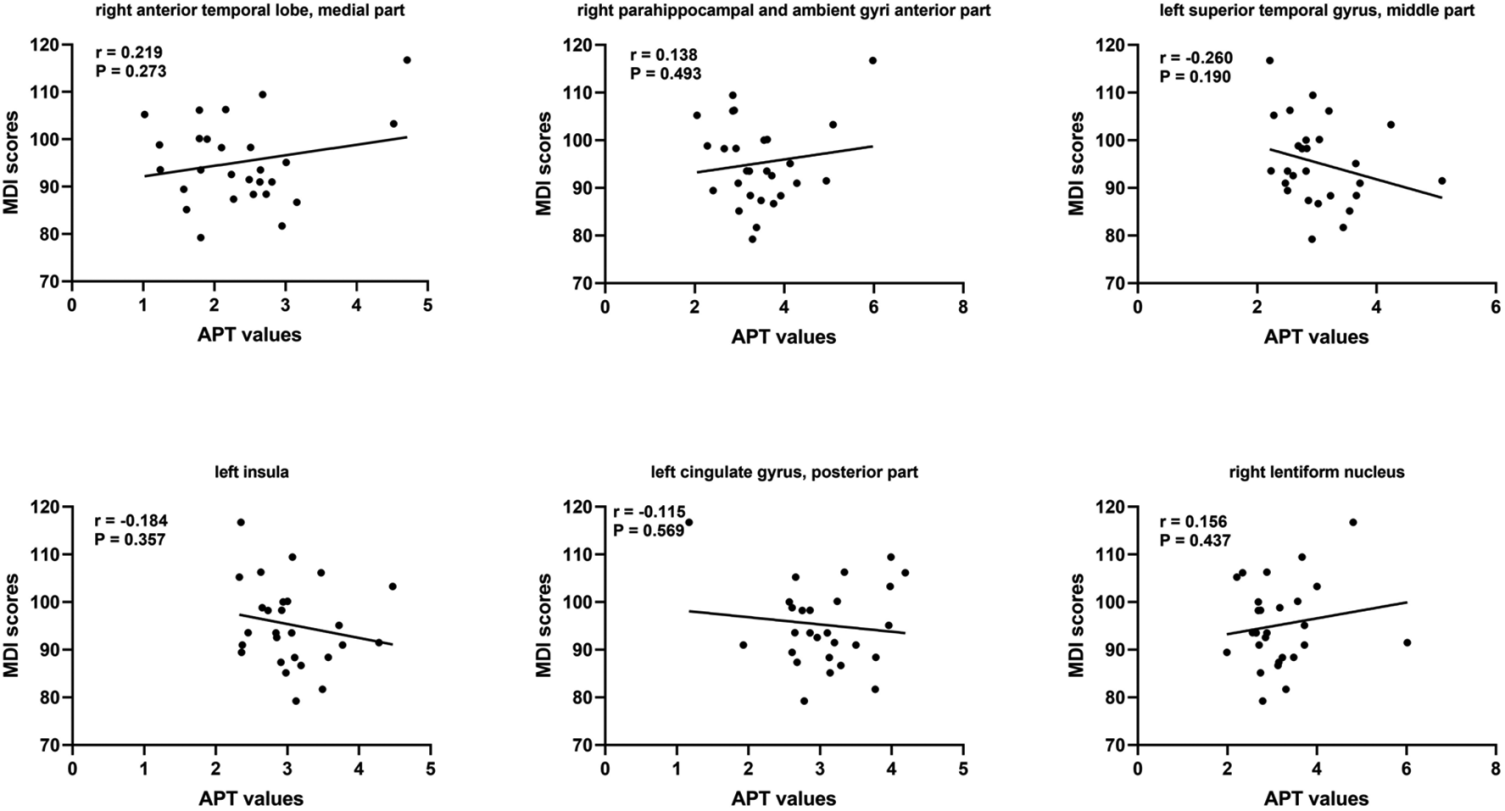
Relationship between MDI scores and region APT values in HIE/MRI+ group.

**Figure 4:**
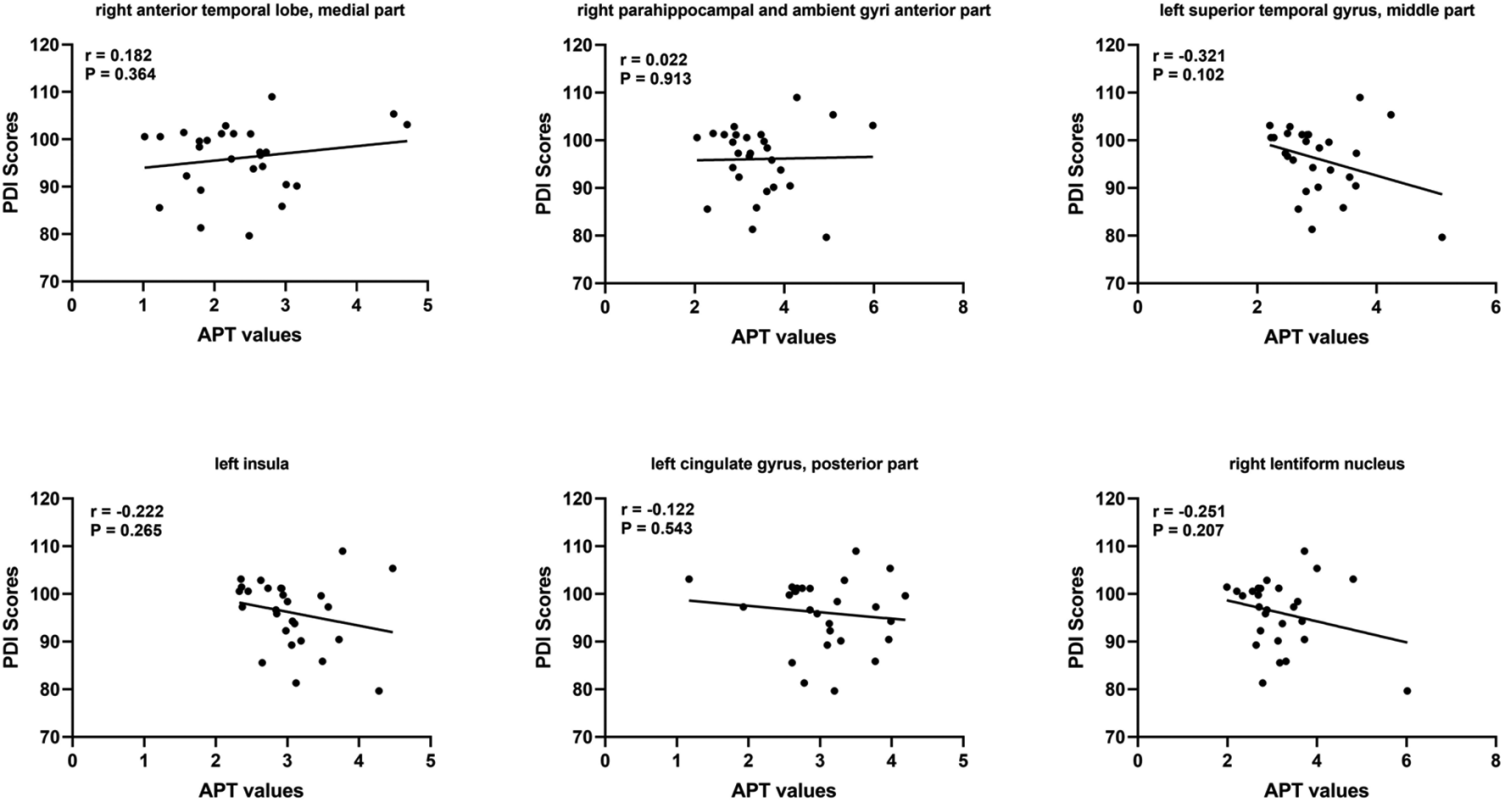
Relationship between PDI scores and region APT values in HIE/MRI+ group.

At short–term neurodevelopmental follow-up, all neonatal have developmental scores with the normal range. But we found the Mental Development Index (MDI) scores and Psychomotor Development Index (PDI) scores had significantly differences among the three groups. Further comparisons were found significantly decreased MDI and PDI scores in HIE/MRI+ group (95.20 ± 8.81, 95.83 ± 7.13, respectively), when compared with controls (108.21 ± 4.85, 107.87 ± 6.56, respectively) and HIE/MRI− group (107.15 ± 4.50, 101.22 ± 7.67, respectively). And there were significant PDI scores differences between HIE/MRI− and control groups (101.22 ± 7.67 vs. 107.87 ± 6.56). The outcome data was showed in Figure 2. We did not find significant correlations between regional brain APT values and MDI or PDI scores in HIE/MRI+ group (Figures 3, 4).

## Discussion

APT imaging is a type of MRI technique that detects *in vivo* pH levels and protein concentrations in tissues in a relatively non-invasive manner on the strength of proton exchange between proteins and free water. The basic pathophysiological processes of HIE in neonates are brain hypoxia and hypoperfusion leading to the occurrence of a series of events, including acidosis, the release of inflammatory mediators, and the formation of free radicals. In the initial stage of hypoxia–ischemia, decreased cerebral blood flow alters energy metabolism, causing anaerobic metabolism (16). As a result, the production of lactic acid increases, followed by cellular acidosis (17). We consider that the protein concentration in the brain is constant over a short duration of time, while the process of proton exchange between saturated amides and water is mainly affected by the pH (18).

When anaerobic conditions persist, damage to the brain tissues is associated with the loss and degradation of some proteins and peptides, as well as the changes in pH (19). Currently, simple MTRasym analysis cannot distinguish the effect of pH from protein information. Whereas, the direction of the APT effect is matching. Therefore, regardless of whether only the pH is lowered or both the pH and protein content are decreased, the exchange rate between saturated amide protons and water protons decreases. This is the foundation of APT imaging as applied to HIE (20).

We know the commonest hypoxic ischemic injury patterns in full–term neonate involve either injury to the basal ganglia and thalamus, or to the watershed region, including the parasagittal cortex and subcortical white matter. In this work, cerebral APT SI changes were detected in multiple brain regions involve deep grey nuclei and watershed region in HIE infants, regardless of conventional MRI findings. Specifically, increased cerebral APT SIs were detected in the left insula, right occipital lobe, left cingulate gyrus (posterior part), and corpus callosum in the HIE/MRI− group compared with controls. This may be related to the mostly infants with HIE were mild in HIE/MRI− group. The patterns of injury in mild HIE can be subtle and are more likely to involve watershed zones and white matter injury including punctate white matter lesions, rather than injury to the deep central gray matter. A recent study applying perfusion–weighted imaging and histopathology demonstrated that the Papez circuit in neonates is also vulnerable to hypoxic–ischemic injury (21). In an HIE animal model, Zheng and Wang (22) showed that APT SIs initially tended to decrease rapidly and then increased after the occurrence of hypoxic–ischemic brain injury. They assumed that protein content and temperature remained unchanged for a short time after the brain insult, and the alteration of APT signals could be primarily related to the intracellular change in pH in brain tissues. Experiments performed in a rat model (23) reported that Lac levels increased at 0–2 and 24 h after the onset of HIE. However, studies have shown pH decreases transiently following hypoxic–ischemic brain injury and then increases, which induces rebound alkalosis (24). During hyperacute, acute, and subacute cerebral hemorrhage, APT signals are significantly higher. This could explain why cerebral APT SIs were increased in the HIE group in this study. With regard to increased APT SIs in HIE infants, it would be worthwhile exploring if this represents progression to permanent injury given that these regions have been known to be susceptible to hypoxic–ischemic brain injury.

In the HIE/MRI− group, no signal changes in conventional MRI were observed, including DWI. However, significantly increased APT SIs were detected in the left insula, right occipital lobe, left cingulate gyrus (posterior part), and corpus callosum compared with controls. In a rat model study of HIE have found that CEST MTR maps did not correspond with the areas of hyper intensity on DWI at 0–2 and 24 h after the onset of HIE. Studies on ischemic stroke have shown that APT-abnormal areas are larger than those with hyperintensity on DWI images. Such discrepancy shows that the effect of APT is different from that of the mechanism generating signals in DWI. Indeed, while APT imaging reflects the pH change in the lesion, DWI imaging reflects the extent of cytotoxic edema. In short, both modalities respectively examine distinct tissue characteristics. Therefore, APT imaging can be used to observe *in vivo* metabolite changes caused by hypoxia–ischemia that cannot be detected by DWI. The combination of APT imaging and traditional imaging modalities can provide clinicians with more diagnostic information.

No significant APT SI differences were observed in the brainstem, cerebellum, among the three groups. It may be due to intrinsic vascular autoregulation that maintains perfusion to these cerebral regions. It can protect them in mild to moderate hypoxic–ischemic brain injury (25) found in most of the existing subjects. An animal study showed that episodes of prolonged fetal hypoxia caused shunting of blood to important brain structures (such as the brainstem, cerebellum, and basal ganglia), at the cost of less metabolically active structures (i.e., the cerebral cortex and white matter) (26). However, what calls for special attention is that the occurrence of prolonged, severe hypoxic–ischemic events leading to permanent injury to deep structures is correlated with poor outcomes (27).

We have observed that all neonatal have developmental scores with the normal range, but neonatal with HIE/MRI+ have significantly lower MDI and PDI scores measured with the Bayley-III compared with healthy control group and HIE/MRI− group. This is mainly because 82.5% (33/40) of neonatal with HIE are mild. Previous research shown that children with mild HIE have normal developmental outcomes. However, there are also studies shown that children with mild HIE are not following the same developmental trajectory as their peers (28–30). Therefore, whether those identified brain structures are associated with mental and motor functions, longer follow-up is needed to investigate how these subtle disabilities will influence the school–age performance of these children. In addition, we did not demonstrate significant correlation region APT values the in prediction HIE outcomes. However, this remains to be proven in further studies.

This study also had limitations. First, HIE cases were selected based on clinical diagnosis by respective treating physicians, and were mainly mild, while moderate and severe HIE cases were relatively less represented. Second, this study adopted a cross–sectional design with short–term neurodevelopmental follow-up. These findings need to be further verified in a cohort of patients with long–term neurodevelopmental follow-up. Third, it can be challenging to delineate anatomic boundaries during segmentation, particularly in neonatal individuals. However, this automatic segmentation approach is more accurate than manual two–dimensional slice annotation. Lastly, APT data of the neonatal brain have low resolution, which may raise questions about the validity of APT SIs, particularly in the cranial base structure. Therefore, we believe that study robustness will be further improved in the future.

## Conclusion

In summary, we demonstrated that cerebral APT SI changes can be detected in HIE patients, including those with normal conventional MRI findings, highlighting the value of APT imaging in the evaluation of suspected HIE. APT SI changes may provide meaningful insights into the characterization of the brain's internal environment. Therefore, APT imaging is a promising tool for early risk stratification of neonatal HIE and could be a helpful complement to conventional MRI in the clinical evaluation of HIE.

## Data Availability

The raw data supporting the conclusions of this article will be made available by the authors, without undue reservation.
